# Editorial: Environmental endocrine disruptors: mechanisms, implications, and advances in detection and mitigation in endocrinology

**DOI:** 10.3389/fendo.2024.1510917

**Published:** 2024-11-06

**Authors:** Qiong Wu, Hongda Liu, Rui Zhang, Xiaolei Zhang, Pu Xia

**Affiliations:** ^1^ School of Water Conservancy and Environment, University of Jinan, Jinan, China; ^2^ Department of General Surgery, First Affiliated Hospital of Nanjing Medical University, Nanjing, China; ^3^ Department of Urology, First Affiliated Hospital of Nanjing Medical University, Nanjing, China; ^4^ Environmental Genomics Group, School of Biosciences, University of Birmingham, Birmingham, United Kingdom

**Keywords:** endocrine disruptors, endocrine dysfunction, systemic diseases, infertility, diabetes

Endocrine disruptors (EDs) are a group of exogenous chemicals that adversely affect human health by altering the structure and function of the endocrine system (1). EDs can induce a wide range of endocrine-related diseases, such as breast cancer, infertility, precocious puberty in children, obesity, diabetes and neurobehavioral abnormalities, among which, many belong to systemic diseases (2). Moreover, there’s a growing consensus that exposure to EDs, even at low levels, also have profound adverse effects, especially during critical windows of development (3). With the incidence of such diseases increasing globally over the past few decades, understanding the roles of EDs underlying the genesis and progression of various systemic diseases becomes crucial. In addition, the research on environmental sources of EDs, mechanisms of action, and potential molecular, cellular and epigenetic changes induced by exposure to EDs is warranted, which can provide insights into the innovative preventive and therapeutic strategies of EDs-related systemic diseases.

Therefore, this Research Topic aims to feature Original Research articles and Reviews which critically explore the multifaceted relationships between EDs and endocrine-related adverse effects, especially systemic diseases. A series of articles have been published under this Research Topic, as shown below.

Organophosphate esters (OPEs) are a group of widely used chemical flame retardants with potential biotoxicity. Animal experiments and epidemiological studies have found that OPEs may interfere with thyroid function (4). However, the association between OPEs and thyroid diseases remains unclear. Therefore, Lin et al. explored the relationships between urinary OPE metabolites and thyroid disease risk in the general population in the United States based on the National Health and Nutrition Examination Survey using Weighted Quartile Sum regression and Bayesian Kernel-Machine Regression models. This study revealed a significant association between OPE metabolites and increased risk of thyroid disease, with bis(2-chloroethyl) phosphate (BCEP) as the predominant contributor. Moreover, the risk of thyroid disease initially decreased and then increased with increasing levels of BCEP, which exhibited a J-shaped pattern.

Platelet-rich plasma (PRP) is a substance derived from a person’s own blood that contains a high concentration of platelets and exhibits minimal immune rejection (5). Using platelets to stimulate cell proliferation and tissue differentiation has emerged a promising approach in regenerative medicine recently. PRP has shown significant potential in promoting endometrial hypertrophy and follicular development, making it a promising treatment option for repairing or replacing damaged tissues or organs. Recent clinical studies have used PRP to treat the female reproductive system and have yielded impressive results. Therefore, Wang et al. provides an overview of the recent advancements, clinical trials and applications, underlying mechanisms, advantages, shortcomings and possible clinical challenges of PRP therapy for various female reproductive and endocrine diseases. They finally called for more research to be conducted to fully understand the promising therapeutic approach and establish evidence-based guidelines for its use.

Bisphenol A (BPA) is a signature ED that severely disrupts the human endocrine system, leading to reproductive and developmental disorders. Given the inter-regulatory relationship between sex hormones and gut flora (6), Wang et al. proposed the ‘gut-liver-hormone axis’ on the basis of the gut-liver axis to investigate subchronic toxic effects of BPA in rats via metagenomic and metabolomic analyses as well as histopathological and biochemical evaluations. The metagenomic results showed that BPA exposure reduced the diversity of intestinal flora. Metabolomic results indicated that BPA altered the levels of some small molecule metabolites, which were closely associated with the gut-liver-hormone axis, such as bile acids and short-chain fatty acids. Histopathological and biochemical analyses suggested that exposure induced hepatic immune inflammatory responses and pathological damage in the heart, liver and testis. Overall, this study provided a novel perspective on the endocrine-related toxic mechanisms of BPA in rats, i.e., via disrupting the gut-liver-hormone axis.

The etiology of diabetes and its complications is complex and not fully understood. An increasing number of studies show that exposure to EDs increases the incidence of diabetes (7). He et al. reviewed the latest epidemiologic and pathogenic evidence on the relationship between EDs and diabetes and its complications. After analysis, they pointed out that mitochondrial dysfunction induced by EDs, including disruption of the electron transport chain, perturbation of Ca^2+^ homeostasis, increased production of ROS, and activation of mitochondrial apoptotic pathways, may be the key mechanism underlying increased incidence rate of diabetes and its complications induced by exposure to EDs. They also call for more animal and cytological studies in the future to complement this observation and provide deeper insights into the mechanisms involved.

Neonicotinoids are one of the most commonly used pesticides worldwide, which were designed to target the nicotinic acetylcholine receptors in insect selectively and disturb the central nervous system, leading to insect paralysis and death. However, rising evidence revealed that neonicotinoids exhibited a number of additional toxicities such as mitochondrial dysfunction, endocrine disruption, reproductive toxicity and immunotoxicity to non-target organisms (8, 9). Currently, the endocrine-disrupting effects of neonicotinoids on important aquatic insects Chironomidae following long-term exposure remains unknown. Therefore, Wei et al. investigated the endocrine-related ecdysis and sex ratio, along with relevant gene expressions of *Chironomus kiinensis* following exposure to a representative neonicotinoid dinotefuran at different environmentally relevant concentrations. The results demonstrated that low-dose dinotefuran downregulated gene expressions related to ecdysis hormones, and delayed growth and development via inhibiting ecdysis. Moreover, it shifted sex ratios toward male-dominated populations. These findings can improve our understanding of the endocrine-disrupting mechanisms and risks of neonicotinoid insecticides in Chironomidae.

In summary, the articles collected under this Research Topic highlight and elucidate the adverse effects of several typical and novel environmental EDs and their potential mechanisms of toxicity and pathogenesis, as well as a promising therapeutic approach for reproductive and endocrine diseases. This Research Topic has positive implications for deepening the understanding of the impact of EDs on endocrine-related adverse effects and diseases, and developing innovative preventive and therapeutic strategies of EDs-related diseases.
